# Phylogenetic Occurrence of the Phenylpropanoid Pathway and Lignin Biosynthesis in Plants

**DOI:** 10.3389/fpls.2021.704697

**Published:** 2021-08-17

**Authors:** Tao Yao, Kai Feng, Meng Xie, Jaime Barros, Timothy J. Tschaplinski, Gerald A. Tuskan, Wellington Muchero, Jin-Gui Chen

**Affiliations:** ^1^Biosciences Division, Oak Ridge National Laboratory, Oak Ridge, TN, United States; ^2^Center for Bioenergy Innovation, Oak Ridge National Laboratory, Oak Ridge, TN, United States; ^3^Biology Department, Brookhaven National Laboratory, Upton, NY, United States; ^4^BioDiscovery Institute and Department of Biological Sciences, University of North Texas, Denton, TX, United States

**Keywords:** lignin biosynthesis, tracheophytes, transcription factor, EPSP synthase, phylogenetic occurrence, lignin utilization

## Abstract

The phenylpropanoid pathway serves as a rich source of metabolites in plants and provides precursors for lignin biosynthesis. Lignin first appeared in tracheophytes and has been hypothesized to have played pivotal roles in land plant colonization. In this review, we summarize recent progress in defining the lignin biosynthetic pathway in lycophytes, monilophytes, gymnosperms, and angiosperms. In particular, we review the key structural genes involved in *p*-hydroxyphenyl-, guaiacyl-, and syringyl-lignin biosynthesis across plant taxa and consider and integrate new insights on major transcription factors, such as NACs and MYBs. We also review insight regarding a new transcriptional regulator, 5-enolpyruvylshikimate-3-phosphate (EPSP) synthase, canonically identified as a key enzyme in the shikimate pathway. We use several case studies, including EPSP synthase, to illustrate the evolution processes of gene duplication and neo-functionalization in lignin biosynthesis. This review provides new insights into the genetic engineering of the lignin biosynthetic pathway to overcome biomass recalcitrance in bioenergy crops.

## Introduction

It is hypothesized that the first land plants possessed adaptive metabolic, physiologic, and morphologic changes as a means of coping with abiotic stresses, such as UV-B irradiation and desiccation (Niklas et al., 2017). In this scenario the phenylpropanoid pathway played a pivotal role in land colonization of early plants by yielding protective secondary metabolites including flavonoids and lignin. Many flavonoids bestowed land plants with the ability to absorb UV-B, while lignin, as the cell wall component, provided mechanical support and facilitated water transport for the vascular plants (Rensing, 2018). Recently several comparative genomics, phylogenetics, and evolutionary genetics approaches have been employed to illustrate the evolution of phenylpropanoid biosynthetic pathway (Ma and Constabel, 2019; Davies et al., 2020). In this review, we unite these current outcomes and provide a comprehensive overview of the phylogenetic occurrence of phenylpropanoid biosynthetic and lignin biosynthetic pathways and showcase the role of gene duplication and neo-functionalization contributing to land plant evolution.

To aid our understanding of the phylogenetic occurrence of the phenylpropanoid pathway and lignin biosynthesis in plants, we offer a primer on lignin biosynthesis. Lignin is derived from three major hydroxycinnamyl alcohols, including *p*-coumaryl alcohol, coniferyl alcohol, and sinapyl alcohol by radical coupling (Weng and Chapple, 2010). As such, *p*-hydroxyphenyl (H), guaiacyl (G), and syringyl (S) monolignols are the main units for lignin polymerization. In addition, two additional non-canonical monolignols, caffeyl alcohol (C), and 5-hydroxyconiferyl (5HG) alcohol, have been found naturally in some species or can be introduced *via* genetic engineering (Dixon and Barros, 2019; Wang X. et al., 2020).

The lignin biosynthetic pathway has been refined and re-envisioned by several research groups over the past two decades. Based on recent studies in the model herbaceous plant *Arabidopsis* and the model woody plant *Populus*, eleven core structural enzymes of the lignin biosynthetic pathway have been identified (Boerjan et al., 2003; Vanholme et al., 2013; Zhang et al., 2020). L-phenylalanine ammonia-lyase (PAL), 4-hydroxycinnamate CoA ligase (4CL), and cinnamate 4-hydroxylase (C4H) are the three enzymes that belong to the general phenylpropanoid pathway shared by the biosynthesis of lignin and flavonoids. Generally, the initial substrate of the phenylpropanoid pathway, phenylalanine, is converted into cinnamate by PAL, C4H coverts cinnamate into *p*-coumarate, and *p*-coumarate is then activated by 4CL to form *p*-coumaroyl CoA.

**FIGURE 1:**
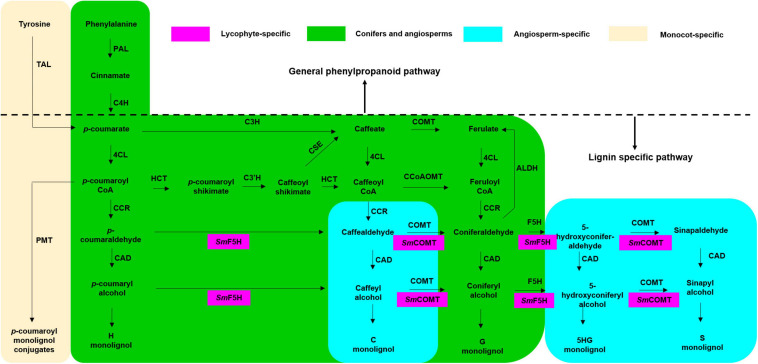
Schematic representation of pathways to produce H, G, and S-monolignols across different plant species. Pathways leading to monolignol biosynthesis include general phenylpropanoid pathway and lignin-specific pathway, which are separated by the dash line. S-lignin biosynthesis in lycophytes occurs in an independent pathway mediated by SmF5H and SmCOMT (highlighted in purple). Some pathways are shared by conifers and angiosperms (highlighted in green) while the others are angiosperm-specific (highlighted in blue). There is also monocot-specific pathway mediated by TAL (highlighted in yellow).

The other eight enzymes belong to lignin-specific pathway (Figure 1), including cinnamoyl CoA reductase (CCR), cinnamyl alcohol dehydrogenase (CAD), coumarate 3-hydroxylase (C3H), coumaroyl shikimate 3′-hydroxylase (C3′H), ferulate/coniferaldehyde 5-hydroxylase (F5H), caffeate/5-hydroxy-coniferaldehyde 3/5-O-methyltransferase (COMT), caffeoyl CoA 3-O-methyltransferase (CCoAOMT), hydroxycinnamoyl CoA: shikimate hydroxycinnamoyl transferase (HCT), and caffeoyl shikimate esterase (CSE). *p*-coumaroyl CoA is converted into the simplest H-lignin monomer by a reductase CCR and a dehydrogenase CAD. In addition to CAD and CCR, G-lignin biosynthesis starting from *p*-coumarate requires C3H, COMT, and 4CL; or 4CL, HCT, C3′H, CSE, and CCoAOMT. F5H and COMT are crucial for S-lignin biosynthesis. Noticeably, aldehyde dehydrogenase (ALDH) catalyzes the opposite direction of reactions in lignin biosynthesis, which is required for ferulate and sinapate biosynthesis from coniferaldehyde and sinapaldehyde, respectively (Nair et al., 2004).

## Lignin Biosynthetic Pathway in Tracheophytes

### The Origin of Lignin Biosynthetic Pathway

Although lignin has not been discovered in bryophytes, nine structural gene families that are responsible for the biosynthesis of H- and G-lignin monomers occur in moss genomes (Xu et al., 2009; Table 1). Studies using the model plant *Physcomitrella patens* shed light on the biosynthetic pathway of phenylpropanoids and lignin. Knock-out of the *CYP98* gene in *P. patens*, which encodes a P450 oxygenase, blocks the biosynthesis of the moss cuticle, thus affecting gametophore formation and organ fusion. C3′H is a homolog of CYP98 in higher plants. However, CYP98 in moss uses the *p*-coumaroyl-threonate as substrate, whereas C3′H in higher plants uses *p*-coumaroyl-shikimate as substrate (Schoch et al., 2001), leading to the distinct biosynthetic pathway for cuticle (Renault et al., 2017). Interestingly, no phenylpropanoid genes have been found in red algae genomes, but trace amounts of lignin have been reported in red algae, and as such, indicating that the lignin biochemical machinery preexisted the evolution of land plants (Martone et al., 2009; Brawley et al., 2017). The extant presence of lignin in red algae may also represent convergent evolution independent of lignin biosynthesis in bryophytes.

**TABLE 1:** Copy number variation of lignin biosynthetic genes in selected model species.

Gene family	*C. reinhardtii* (green algae)	*P. patens* (moss)	*A. thaliana*	*P. trichocarpa*	*O. sativa*
*PAL*	0	26	4	7	9
*4CL*	0	4	4	7	5
*C4H*	0	6	1	4	4
*CCR*	0	1	4	13	14
*CAD*	1	4	9	26	12
*C3H*	0	3	3	3	3
*F5H*	0	0	1	4	3
*COMT*	0	4	1	13	9
*CCoAOMT*	0	5	2	2	5
*HCT*	0	12	1	9	22
*CSE*	0	1	1	2	1
*ALDH*	4	37	14	30	21

### H-Lignin Biosynthesis in Seedless Vascular Plants

During land plant evolution, lignin appeared first in lycophytes (Renault et al., 2019) in the form of H-lignin. Interestingly, there are only low levels of H-lignin in gymnosperms and traces of H-lignin in angiosperms. In contrast, H-lignin is highly abundant in seedless vascular plants, including lycophytes and pteridophytes. Lignin is found between the cellulose matrix and forms a rigid cell wall in these plants (Espiñeira et al., 2011; Ralph et al., 2019). In gymnosperms and angiosperms, H-lignin can be enriched by down-regulation of *C3′H*, *HCT*, and *CSE* genes (Franke et al., 2002a; Wagner et al., 2007; Coleman et al., 2008; Li et al., 2010; Vanholme et al., 2013, Fornalé et al., 2015), though in many cases growth was negatively impacted. Interestingly, the *Arabidopsis C3′H* mutant *ref8* showed severe growth defect that was rescued by disruption of the mediator complex units MED5a and MED5b (Bonawitz et al., 2014). These results indicate that H-lignin may represent one of the earliest forms of lignin.

### G- and S-Lignin Biosynthesis in Pteridophytes

G-lignin biosynthesis in pteridophytes is evolutionarily conserved. The *Df4CL2* gene is a 4-coumarate:coenzyme A ligase coding gene identified from the fern species *Dryopteris fragrans*. Heterologous expression of this gene in tobacco increased the synthesis of lignin, demonstrating the conserved function of *4CL* in *D. fragrans* and tobacco (*Nicotiana tabacum*) (Li et al., 2020). Similarly, two *CCoAOMTs* have been cloned from the fern species *Polypodiodes amoena*, and their functions in lignin biosynthesis have been confirmed *via* heterologous expression in *Arabidopsis* (Zhang X.-S. et al., 2019).

S-lignin has been identified in lycophytes such as *Selaginella moellendorffii*; however, its biosynthetic pathway is different from that in angiosperms (Renault et al., 2019). In angiosperm, both *C3H* and *F5H* are involved in S-lignin biosynthesis. In contrast, in lycophytes, *SmF5H* has dual functions that enables S-lignin to be synthesized directly from *p*-coumaraldehyde and *p*-coumaryl alcohol. Here, *SmF5H* and *SmCOMT* form a gene cluster and are responsible for S-lignin biosynthesis. Phylogenetic analyses suggest that these two genes were independently evolved from their counterparts in angiosperm (Weng et al., 2008b, 2011). Besides the well-known S-lignin biosynthesis in *Selaginella*, several ferns, such as *Dennstaedtia bipinnata*, also contain a large amount of S-lignin in the sclerotic sheaths. However, the biosynthetic pathway has not been elucidated (Logan and Thomas, 1985; Weng and Chapple, 2010). Further studies of the lignin biosynthesis-related genes in these fern species and comparation with what we have known in other species in the lineage is needed to provide insights on the evolution of the S-lignin biosynthetic pathway.

### G-Lignin Biosynthesis in Gymnosperms

Gymnosperms diverged from angiosperms 300 million years ago (De La Torre et al., 2020). In general, gymnosperms lack the *F5H* gene, and therefore, gymnosperm lignin mainly contains G-monolignol and contains no or little S-monolignol (Li et al., 2001; Weng and Chapple, 2010). When *Cf4CL* and *CfCCoAOMT* were cloned from *Cryptomeria fortunei*, a gymnosperm, and heterologously expressed in tobacco, an angiosperm, G-lignin biosynthesis was increased, indicating that these two lignin genes can function equally well in both gymnosperms and angiosperms (Guo et al., 2019). Similarly, a CSE, LkCSE, from *Larix kaempferi*, can convert caffeoyl shikimate to caffeate and shikimate, supporting the conserved function of CSE between gymnosperms and angiosperms (Wang et al., 2019). Gymnosperms also produce a compression layer within xylem that enriched in H-lignin in tracheid. A recent study showed that spatial patterning of H- and G-lignin during wood formation is related to different localizations and enzyme activities of lignin polymerization enzymes, laccases (Hiraide et al., 2021). Interestingly, some gymnosperm species, such as *Gentales*, can also synthesize S-lignin (Renault et al., 2019). *Gnetum genmon* contains angiosperm-like vessels as well as tracheids and fiber tracheids (Tomlinson, 2001), and it shares the chemical compositions of lignin with angiosperms (Nawawi et al., 2016). These results suggest that the biosynthetic pathway for G-lignin is shared between gymnosperms and angiosperms. On the basis of these results we are left with two alternate hypotheses; ancient gymnosperms were able to produce S-lignin which was subsequently lost in modern gymnosperms or the occurrence of S-lignin in *Gentales* is a recent convergent evolutionary event. As an ancient gymnosperm, further systematic studies of lignin biosynthesis in *Gentales* are needed to definitively describe the evolution trajectory in gymnosperms.

### G- and S-Lignin Biosynthesis in Angiosperms

Angiosperms contain the lignin composed of G-, S-, and H-lignin monomers in various ratios (Mansfield et al., 2012). The lignin biosynthetic pathways of angiosperms have been characterized using the model plants, *Arabidopsis*, *Populus*, and *Brachypodium*, among others. Xu et al. (2009) analyzed 10 of 11 lignin biosynthetic gene families (without CSE) across 14 plant species and 1 symbiotic fungal species using comparative genomics. The analysis revealed that the rapid expansion of these gene families occurred after the divergence between dicots and monocots 140–150 million years ago (Xu et al., 2009; Rao and Dixon, 2018).

Although the lignin biosynthetic pathways are generally conserved among angiosperms, alternative pathways have evolved in monocots. In dicots, the first enzyme in the phenylpropanoid pathway, PAL, converts phenylalanine (Phe) to cinnamate. Cinnamate is then converted to *p*-coumarate by the second enzyme, C4H. However, a bypass route has been discovered in monocots. PTAL was identified as a bifunctional enzyme that recognizes tyrosine (Tyr) as the substrate and converts it to *p*-coumarate directly in *Brachypodium distachyon* (Barros et al., 2016). ^13^C isotope feeding with *BdPTAL1-RNAi* transgenic plants revealed that BdPTAL1-mediated lignin biosynthesis contributed to half of the total lignin content in *B. distachyon* (Barros et al., 2016). Another grass-specific enzyme is *p*-coumaroyl-CoA:monolignol transferase (PMT) that catalyzes the incorporation of *p*-coumarate into the lignin polymer backbone typically found in the Poaceae family (Withers et al., 2012; Petrik et al., 2014). These findings suggest that lineage-specific lignin biosynthetic pathways have evolved independently in dicots and monocots and highlight the need to study species-specific branches in the lignin biosynthetic pathway.

The *C4H* gene progenitor appears to have duplicated in early seed plants, yielding two clades that are preserved in Taxaceae and most angiosperms. A second duplication event happened after the divergence of dicots and monocots. By analyzing the protein structure and function of *Brachypodium* C4H, it was found that each of *Brachypodium C4H* paralog genes can rescue the growth defect of the *Arabidopsis c4h* mutant, indicating that the *C4Hs* in monocots preserved the canonical function in lignin biosynthesis. However, the protein structures of C4Hs in *B. distachyon* differ from that in *Arabidopsis*. This newly derived C4H type in monocots has an elongated N-terminus, which alters the subcellular localization and allows the orientation of C4H to the lumen of endoplasmic reticulum (ER) through a double-spanning hairpin structure. Therefore, it is possible that an alternate C4H exists within the ER (Renault et al., 2017).

Coumarate 3-hydroxylase and C3′H catalyze the conversion of *p*-coumarate and *p*-coumaroyl shikimate into caffeate (*via* a bifunctional cytosolic ascorbate peroxidase, Barros et al., 2019) and caffeoyl shikimate (*via* a cytochrome P450 monooxygenase, Schoch et al., 2001), respectively. These enzymes play important roles in G-lignin and S-lignin biosynthesis. There is only one member of the *C3′H* family in *Arabidopsis thaliana* and two members of the cytosolic *C3H* family in *A. thaliana* and *B. distachyon* (Franke et al., 2002b; Barros et al., 2019). *PtrC3′H3* was recognized as the homolog of *Arabidopsis* C3′H. However, it was proposed that PtrC3′H3 requires PtrC4H1 or PtrC4H2 to form a complex to enhance its enzymatic activity in *Populus trichocarpa* (Chen et al., 2011; Figure 1). Recent study showed that triple knocking-down *PtrC4H1*/*PtrC4H2*/*PtrC3′H3* causes monolignol benzoate (ML-BL) conjugation and significantly reduces lignin biosynthesis while increasing H-lignin for about 70-fold (Kim et al., 2020). These findings suggest that simultaneous modification of *C4H* and *C3H* could be used for reducing biomass recalcitrance in bioenergy crops.

Phylogenetic analysis of 192 *4CLs* across land plants suggested that a duplication of the *4CL* gene family occurred prior to the split of gymnosperms and angiosperms (Li et al., 2015). Functional divergence of the *4CL* gene family, post duplication, has been broadly found in angiosperms. In fact, four members of the *4CL* gene family have been reported in *P. patens*, but only three of them were expressed under tested conditions (Silber et al., 2008). There are four *4CL* genes in *Arabidopsis*, five in rice, and seven in *Populus*. Functional analysis of these gene families revealed that only one subgroup of this gene families is involved in lignin biosynthesis, while other subgroups are involved in the biosynthesis of flavonoids or phenolics *via* neofunctionalization (Ehlting et al., 1999; Gui et al., 2011; Li et al., 2015; Rao et al., 2015; Table 1). Loss-of-function mutation of *4CL* genes in herbaceous species causes reductions in G-lignin and increase of S/G ratios. However, knock-out *4CL1* gene in *Populus* led to reduction of S-lignin and decrease of S/G ratio, and the homeostasis of G-lignin was maintained by *4CL5* in *4cl1* mutant. These findings point toward a functional divergence of *4CLs* between herbaceous and woody species (Xiong et al., 2019; Tsai et al., 2020).

Hydroxycinnamoyl transferase catalyzes the conversion of caffeoyl shikimate to caffeoyl-CoA. Down-regulation of *AtHCT* caused the reduction of S-lignin content in *Arabidopsis* (Hoffmann et al., 2004). The orthologs of *HCT*s are present among all the land plants, which suggests that this enzyme evolved before the occurrence of lignin. A recent study showed that *P. patens HCT* and *Marchantia polymorpha HCT* can complement the deficiency of *Arabidopsis hct* mutant in terms of morphology and metabolite levels, suggesting that the function of *HCT* is likely conserved in all embryophytes (Kriegshauser et al., 2021). It appears that gene duplication of *HCT* occurred in dicots that produced the *HQT* gene. Despite the sequence similarity between *HCT* and *HQT*, the latter is required for biosynthesizing chlorogenic acid rather than lignin in *Cynara cardunculus* (Sonnante et al., 2010). Knock-down of *HCT* led to increase of G-lignin and decrease of S-lignin and S/G ratio in *Populus* (Zhou et al., 2020). However, knock-down of both *HCT1* and *HCT2* did not drastically change lignin content or composition in *B. distachyon*. Meanwhile, the saccharification efficiency was greatly enhanced in the double knock-down line (Serrani-Yarce et al., 2021). These findings suggest *HCT* genes play different roles in some monocots compared to that of dicots.

Caffeoyl shikimate esterase is a newly discovered enzyme involved in monolignol biosynthesis. Together with 4CL, these two enzymes form a bypass pathway of monolignol biosynthesis in *Arabidopsis* (Vanholme et al., 2013). *CSE* genes cloned from *Medicago truncatula* and *Populus deltoides* have been shown to be functionally conserved with their *Arabidopsis* homolog (Ha et al., 2016; Saleme et al., 2017). However, the homolog of *CSE* gene has not been identified in most monocots, including maize and *Brachypodium*. Recently, the generation of *cse1*, *cse2* single mutant and *cse1/cse2* double mutant in *Populus* further confirmed their partial redundant roles in lignin biosynthesis. In addition to causing a 35% reduction in lignin content, the *cse1*/*cse2* double mutant significantly improved cellulose-to-glucose transformation efficiency. As such, *CSEs* in *Populus* could be promising target genes in biorefinery although their growth penalty should be managed to avoid (de Vries et al., 2021). Noticeably, *CSE* has also been shown to be functional in gymnosperms, such as *Larix kaempferi* (Wang et al., 2019). These findings suggest that *CSE* may be evolved prior to the divergence of gymnosperms and angiosperms, but was lost in many monocots (Wang et al., 2019; Serrani-Yarce et al., 2021).

Caffeate/5-hydroxy-coniferaldehyde 3/5-O-methyltransferase and F5H are two key enzymes required for catalyzing the intermediates in G-lignin biosynthesis into S-lignin biosynthesis. It has been reported that simultaneously manipulating *COMT* and *F5H* resulted in a dramatic change of S-lignin biosynthesis (Wu et al., 2019). COMT and F5H in *S. moellendorffii* appears to have an independent origin compared to that of angiosperms. There are two *F5H* genes in *Arabidopsis* (*AtF5H1/CYP84A1* and *AtF5H2/CYP84A2)*, and only *AtF5H1* has been confirmed to be involved in lignin biosynthesis (Meyer et al., 1998). Similarly, there is one functional *COMT* gene identified among 13 homologous genes in *Arabidopsis* (Raes et al., 2003). In *Populus*, five *F5H* genes have been cloned, and two of them, *PtrF5H1* and *PtrF5H2*, were reported to be involved in lignin biosynthesis. Thirteen members of *COMT* gene family were identified in *P. trichocarpa*, but only *PtrCOMT2* is highly expressed in xylem (Shi et al., 2009; Table 1). The function of F5H was shown to be conserved in monocots, such as *Oryza sativa*. One of three *F5H* genes, *OsCAld5H1*, was reported to greatly affect the S/G-lignin composition *via* over-expression or knock-out (Takeda et al., 2017, 2019). OsCAldOMT1 has been proven to be a functional COMT in rice (Lam et al., 2019). Noticeably, it not only regulates S-lignin biosynthesis, but also controls tricin-lignin biosynthesis. The dual functions of OsCAldOMT1 seems to be specific in grass species (Lam et al., 2019). CCoAOMT, another O-methyltransferase, converts feruloyl CoA to sinapoyl CoA and is required for the conversion of G-lignin into S-lignin. Genetic engineering of this enzyme led to change in G-lignin biosynthesis in *Populus*, alfalfa, *Pinus radiata*, maize, and tobacco (Zhong et al., 2000; Guo et al., 2001; Wagner et al., 2011; Li et al., 2013; Xiao et al., 2020). These studies suggest the function of CCoAOMT is likely to be conserved among all angiosperms and occurred with the advent of the angiosperms.

Cinnamoyl CoA reductase recognizes four types of cinnamoyl-CoAs, including *p*-coumaroyl CoA, caffeoyl CoA, feruloyl CoA, and sinapoyl CoA, and converts them into cinnamaldehydes. Phylogenetic analysis of 146 *CCR* genes of various land plants revealed that *CCR* family contains three classes: *CCR*, *CCR-like*, and *DFR*, and that only the *CCR* class contains *bona fide* lignin biosynthetic genes. All these three classes are distributed across land plants, including *P. patens*, which contains a single functional *CCR* gene. These results suggested that the progenitor *CCR* gene evolved after the advent of lycophytes (Barakat et al., 2011). Still, functional divergence within the *CCR* family has arisen in several species. For example, in *Arabidopsis, AtCCR1* is involved in lignin biosynthesis, whereas *AtCCR2* is involved in pathogen response (Lauvergeat et al., 2001; Ruel et al., 2009). Downregulation of a *CCR* gene, *CCR2*, reduces lignin biosynthesis and increases saccharification efficiency in *Populus*. However, it also causes severe biomass penalty (Van Acker et al., 2014). Recently, a *ccr2* mutant was generated by the CRISPR/Cas9 approach that contain a null and haplo-insufficient allele in *Populus*. This mutant line does not have growth penalty, but still has low lignin content and improved saccharification efficiency (De Meester et al., 2020). Therefore, *CCR2* gene could be a useful target that can be deployed in genetic engineering of bioenergy woody crops.

Cinnamyl alcohol dehydrogenase catalyzes the final step of monolignol biosynthesis leading to compositional differences in lignin forms. Guo et al. (2010) performed phylogenetic analysis of the *CAD* gene family from 52 species and classified them into three classes. Class I comprises *bona fide CADs* which are only present in vascular plants, suggestive of their co-occurrence with the advent of lignin. The functional characterizations of Class II and Class III *CADs* remain unclear (Guo et al., 2010). Within the large gene families, *CADC* and *CADD*, *PtrCAD1* and *OsCAD2* have been reported to be functional *CAD* genes involved in lignin biosynthesis in *Arabidopsis*, rice, and *Populus*. Knock-down or knock-out of these genes resulted in reduced lignin content as well as altered lignin structures (Anderson et al., 2015; Van Acker et al., 2017; Martin et al., 2019). Finally, it was reported that CAD and CCR form an enzyme complex that regulates monolignol biosynthesis in *P. trichocarpa* (Yan et al., 2019).

In summary, as an important branch of the phenylpropanoid pathway, structural genes of the lignin biosynthetic pathway are conserved in most embryophytes. *F5H* and *COMT* contribute to S-lignin biosynthesis and have been hypothesized to have independent origins in *S. moellendorffii* and angiosperms. Gene duplications and gene family expansion of lignin biosynthetic genes in angiosperms have given rise to sub-functionalization and neo-functionalization of the various members, which is consistent with their morphological and functional changes compared with lower plants.

## Transcriptional Regulation of Lignin Biosynthetic Pathway

**FIGURE 2:**
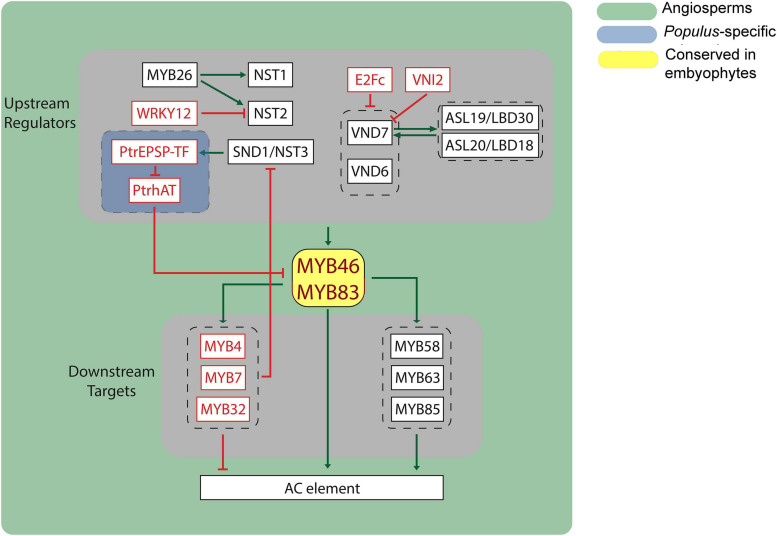
Transcriptional network of lignin biosynthesis in plants. Black box indicates transcriptional activator, and red box indicates transcriptional repressor. Green arrows indicate transcriptional activation, and red blunt arrows indicate transcriptional repression. AC element, recognized by MYBs, were found in most of lignin biosynthetic genes.

The lignin biosynthetic pathway includes both structural genes and regulatory proteins. Transcriptional regulation, controlling the gene expression of structural genes, plays important roles in lignin biosynthesis. Such genes reflect the phylogenetic occurrence of the phenylpropanoid pathway and evolutionary trajectory of lignin biosynthesis in plants. MYBs and NACs are two major transcription factor families, comprising three layers of the hierarchical transcriptional regulatory network (Ohtani and Demura, 2019; Figure 2). Therefore, we focus on analyzing these two families of transcription factors to illustrate the evolutionary divergence of transcriptional regulation in lignin biosynthesis.

### MYB46-Mediated Transcriptional Regulation of Lignin Biosynthesis

Transcription factor MYB46 is a central regulator in secondary cell wall formation (Zhong et al., 2007). MYB46 and MYB83 are two functionally redundant *A. thaliana* MYB transcription factors that act as master switches of lignin biosynthesis regulating nine out of 11 monolignol biosynthetic genes (*PAL*, *C4H*, 4*CL*, *HCT*, *C3′H*, *CCoAOMT*, *F5H*, *CCR*, and *CAD*) (Kim et al., 2014). Besides lignin, the biosynthesis of other secondary cell wall components, including xylan and cellulose, are also regulated by MYB46/MYB83 (McCarthy et al., 2009; Zhong and Ye, 2012; Kim et al., 2013). Several MYB46 orthologs from other plant species have also been shown to function as key regulators for secondary cell wall biosynthesis, including PtMYB4 from pine, EgMYB2 from *Eucalyptus*, OsMYB46 from rice, PtrMYB2, PtrMYB3, PtrMYB20, and PtrMYB21 from *Populus*, and ZmMYB46 from maize (Patzlaff et al., 2003; Goicoechea et al., 2005; Zhong et al., 2011, 2013). The functions of MYB46 and MYB83 in lignin biosynthesis are well-conserved in angiosperms.

The phylogenetic history of lignin related *MYBs* appears to coincide with the advent of the lignin biosynthetic genes, which first emerged in early land plants (Xu et al., 2014; Bowman et al., 2017). Homologs of *MYB46* and *MYB83* have been found in *P. patens* and *S. moellendorffii* (Zhong et al., 2010). Functional conservation of their homologs *via* transgenic validation has also been demonstrated in vascular plants, including gymnosperms and angiosperms (Zhao and Bartley, 2014). We hypothesis that *MYB46* and *MYB83* might be required for phenylpropanoid biosynthesis outside of the lignin biosynthetic pathway in non-vascular plants while playing core roles in lignin biosynthesis in all vascular plants.

### Upstream Regulators of MYB46/MYB83

Major transcription factors regulating *MYB46/MYB83* are the NAC TF family proteins (Figure 2). NAC TF family proteins share a conserved NAC domain located at the N-terminal region and a highly divergent C-terminal activation domain (Olsen et al., 2005). These TFs are specific to plants and play diverse roles in plant defense, growth, and development (Olsen et al., 2005). *NAC SECONDARY WALL THICKENING PROMOTING FACTOR1* (*NST1*) and *NST2* are redundantly responsible for secondary wall thickening in anther endothecium (Mitsuda et al., 2005). A MYB family protein, MYB26, localized in the nucleus, was found to be an upstream positive regulator of *NST1* and *NST2*. Overexpression of *MYB26* was found to increase lignin deposition and the expression of *NST1* and *NST2* (Yang et al., 2007). Recent study shows that Xylem NAC Domain 1 (XND1) interacts with NST1 and inhibits the transcriptional activity of NST1, thus repressing secondary cell formation (Zhang Q. et al., 2019). In addition, VASCULAR-RELATED NAC-DOMAIN 6 (VND6) and VND7 directly regulate *MYB46* and *MYB83* expression (Zhong et al., 2008; McCarthy et al., 2009; Ohashi-Ito et al., 2010; Yamaguchi et al., 2011). Overexpression of *VND6* and *VND7* can induce the ectopic differentiation of metaxylem-like vessels and protoxylem-like vessels, respectively (Kubo et al., 2005). Functional suppression of VND6 and VND7 caused defects in the formation of vessel elements (Kubo et al., 2005; Yamaguchi et al., 2008). In *Arabidopsis*, there are seven *VND* genes (*VND1*-*VND7*). Similar to *VND6* and *VND7*, overexpression of *VND1* to *VND5* also induces ectopic secondary cell wall deposition, suggesting that all VND members contribute to lignin biosynthesis during xylem vessel development (Endo et al., 2014; Zhou et al., 2014).

A third class of TFs involved in lignin biosynthesis include the WRKY gene family. Mutation of the *Arabidopsis WRKY12* gene caused secondary cell wall thickening in pith cells that is associated with ectopic deposition of lignin, xylan, and cellulose. *WRKY12* mutation upregulated the transcription of downstream genes encoding the NAC domain TF NST2 and the zinc finger TF C3H14, which activate secondary wall synthesis (Wang et al., 2010). Direct binding of WRKY12 to the *NST2* gene promoter led to repression of *NST2* and *C3H14*, as defined by *in vitro* assays and *in planta* transgenic experiments (Wang et al., 2010). Interestingly, *WRKY12* gene is expressed in both pith and cortex that do not have secondary wall thickening, suggesting that WRKY12 may control the parenchymatous nature of pith cells by acting as a negative regulator of secondary cell wall NACs (Wang et al., 2010). WRKY15 was reported to repress the expression of *VND7* and suppress tracheary elements (TEs) differentiation through indirect regulation (Ge et al., 2020). Based on our current understanding, WRKY TFs act upstream of NACs to regulate secondary cell wall biosynthesis.

Two members of the ASYMMETRIC LEAVES2-LIKE/LATERAL ORGAN BOUNDARIES DOMAIN (ASL/LBD) family ASL19/LBD30, ASL20/LBD18 were identified to be involved in a positive feedback loop for *VND7* expression that regulates TEs differentiation-related genes (Soyano et al., 2008). Overexpression of *ASL19* and *ASL20* induced trans-differentiation of cells from non-vascular tissues into TE-like cells, similar to those induced by *VND6* or *VND7* overexpression. Expression of both *ASL19/LBD30* and *ASL20/LBD18* are dependent on VND6 and VND7 (Soyano et al., 2008). XND1 has been reported to inactivate VND6 by physically interacting with VND6 and directing VND6 from the nucleus to the cytoplasm (Zhong et al., 2020). Another NAC transcriptional factor, VND-INTERACTING2 (VNI2), can bind to VND proteins and has been shown to function as a transcriptional repressor of VND7-mediated gene transcription (Yamaguchi et al., 2010). Recent studies show that E2Fc is a key upstream regulator of *VND6* and *VND7*, directly targeting the genomic loci of *VND6* and *VND7*. E2Fc is a transcriptional repressor, and transcript abundance of *VND6* and *VND7* were significantly increased in *E2Fc* knockdown *Arabidopsis* lines (Taylor-Teeples et al., 2015). Taken together, VND6 and VND7 represent key regulators in lignin biosynthesis whose functions are tightly regulated by various TFs (Ko et al., 2012; Schuetz et al., 2013). Phylogenetic analysis discovered close homologs of VND6 and VND7 in all vascular plants, whose functions were demonstrated to be conserved in *P. trichocarpa*, *Zea mays*, *Oryza sativa*, and *B. distachyon* (Zhong et al., 2010, 2011; Valdivia et al., 2013).

SND1/NST3 and NST1 are required for secondary wall thickening in stem fibers (Mitsuda et al., 2007). When these genes were expressed constitutively in *Arabidopsis*, ectopic secondary wall thickening in various tissues was induced (Mitsuda et al., 2005, 2007). Putative orthologs of *NST1*, *NST2*, and *SND1/NST3* are present in the genome of *Populus* and are expressed in developing xylem (Mitsuda et al., 2007), implicating a role in lignin biosynthesis. The function of NST homologs in lignin biosynthesis has been confirmed in *Medicago truncatula* and cotton (Zhao et al., 2010; Fang et al., 2020). However, the homologs of NST proteins have not been identified in gymnosperms or earlier species, implying that these proteins may not have evolved until the appearance of angiosperms (Nakano et al., 2015).

### Downstream Targets of MYB46/MYB83

Three MYB family proteins, MYB58, MYB63, and MYB85, whose coding genes are direct targets of MYB46, have been shown to function as direct transcriptional activators of lignin biosynthesis during secondary wall formation in *Arabidopsis* (Zhong et al., 2008; Ko et al., 2009; Demura and Ye, 2010; Zhou et al., 2020; Figure 2). All three MYBs cause ectopic lignin deposition when overexpressed.

The coding genes of three other MYB family proteins, MYB32, MYB4, and MYB7, are also directly activated by MYB46 (Ko et al., 2009). These three MYBs, sharing high sequence similarity with a conserved EAR motif, have been shown to be transcriptional repressors (Dubos et al., 2010). Trans-activation assays showed that these MYB transcription factors directly repress the expression of *SND1*, forming a feedback regulatory loop to maintain the abundance of SND1 (Wang et al., 2011).

KNOTTED ARABIDOPSIS THALIANA7 (KNAT7) and BEL1-LIKE HOMEODOMAIN6 (BLH6) belong to knotted-like homeobox proteins and bel1-like homeodomain proteins, respectively. *KNAT7* and *BLH6* were reported to be direct targets of MYB46 and MYB83 (Zhong and Ye, 2012). KNAT7 and BLH6 interact with each other and negatively regulate lignin biosynthesis while KNAT3 was reported to form heterodimer with KNAT7 to synergistically regulate lignin content and composition (Liu et al., 2014; Qin et al., 2020; Wang S. et al., 2020).

Although the first and second layers of master switches of lignin biosynthesis were shown to be conserved in vascular plants even in early land plants, the targets of MYB46/83 have not been shown to be functionally conserved in lower plants. For example, the close homologs of *MYB58* and *MYB63* failed to be identified in lower tracheophytes (Zhong et al., 2010). In addition, their homologs in switchgrass were found to be mainly involved in flavonoids biosynthesis rather than lignin biosynthesis. A plausible explanation is that wide expansion, promiscuous functionality, and functional diversification of the MYB family across different species have made it difficult to identify the genuine orthologs responsible for lignin biosynthesis regulation (Zhao and Bartley, 2014; Nakano et al., 2015). Furthermore, lineage-specific MYBs may contribute to lignin biosynthesis in different tracheophytes. For instance, MYB75 was found to repress secondary cell wall biosynthesis and activate anthocyanin biosynthesis in dicots but not in monocots (Zhao and Bartley, 2014).

### EPSP as a Transcriptional Repressor

**FIGURE 3:**
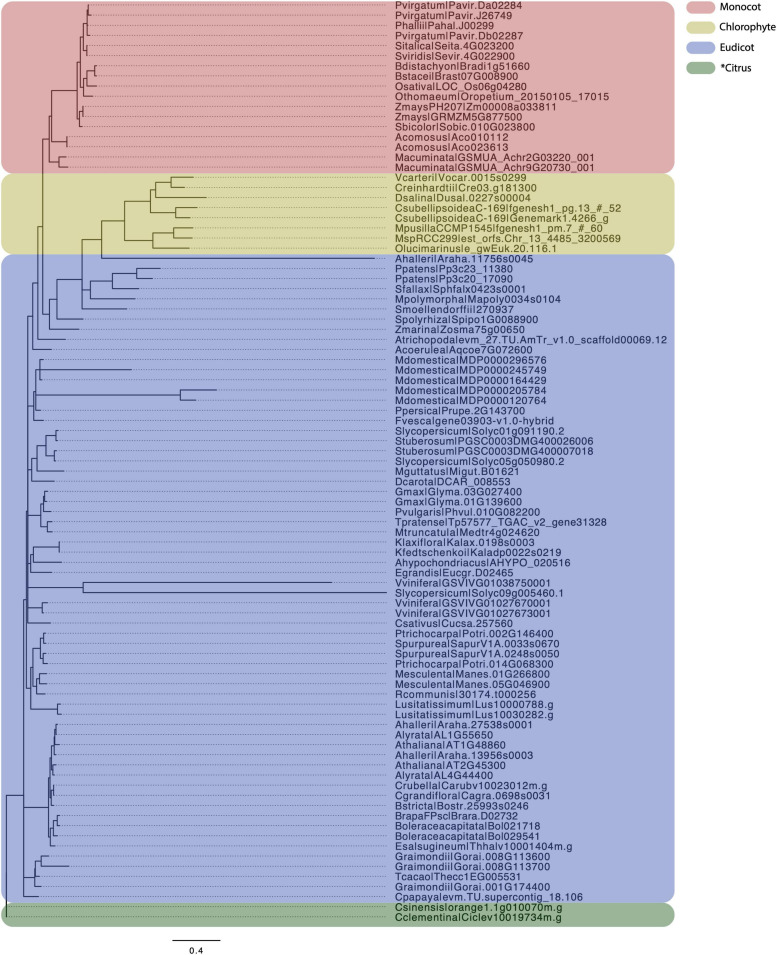
Molecular dating of EPSPs. A total of 91 EPSPs, identified by searching against PtrEPSP-TF in phytozome, were used for molecular dating analysis. We first used MUSCLE (Edgar, 2004) to perform multiple alignments of EPSP proteins, an in-house python script was then used to convert the amino acid alignment to nucleotide alignment, and finally TrimAL (Capella-Gutiérrez et al., 2009) was used to trim the alignment using parameters “-gt 0.8 -st 0.001,” which indicate the tolerating gaps of no more than 20% and similarity score less than 0.001. Mrbayes (Huelsenbeck and Ronquist, 2001) was used to conduct molecular dating with parameters “lset nst = 6 rates = invgamma” using the “GTR + I + Γ” model. A total of 10,000,000 mcmc generations were run after the standard deviation of split frequencies falls under 0.05. FigTree (Rambaut, 2012) was used to visualize the phylogenetic tree. Number of each node indicates the posterior probabilities. Pink, yellow, blue, and green colors separate monocot, Chlorophyte, Eudicot, and Citrus, respectively.

5-enolpyruvylshikimate-3-phosphate (EPSP) synthase is a key enzyme in shikimate pathway, which is present in both plants and many prokaryotes. EPSP synthase has been a well-known herbicide target, which has been widely used in agriculture (Sammons and Gaines, 2014). Noticeably, there is only one copy of an EPSP synthase coding gene in green algae, lycophytes, and bryophytes, but duplicated genes were found in angiosperms, such as *Arabidopsis* and *Populus* (Tohge et al., 2013; Yang et al., 2017; Xie et al., 2018; Figure 3). The gene duplication in angiosperms may have given rise to neo-functionalization for the additional gene copy.

A recent study in *P. trichocarpa* discovered the transcriptional regulatory function of one EPSP synthase gene (*PtrEPSP-TF*) (Xie et al., 2018). Overexpression of *PtrEPSP-TF* led to ectopic deposition of lignin, accumulation of phenylpropanoid metabolites and differential expression of secondary cell wall biosynthetic genes. It was shown that PtrEPSP-TF accumulates in the nucleus and acts as a transcriptional repressor by directly binding to the promoter element of a hAT transposase family gene (*PtrhAT*). PtrhAT is also located in the nucleus and serves as a transcriptional repressor. The direct target of PtrhAT is *PtrMYB021*, which is a homolog of *MYB46* in *Arabidopsis* that acts as a master switch for secondary cell wall biosynthesis, as described above. By repressing the expression of *PtrhAT*, PtrEPSP-TF activates the expression of *PtrMYB021* and the phenylpropanoid pathway (Xie et al., 2018). In conclusion, *PtrEPSP-TF/PtrhAT/PtrMYB021* form an additional regulatory loop in lignin biosynthesis in *Populus*.

PtrEPSP-TF distinguishes itself from ancestral EPSP synthases by carrying an additional helix-turn-helix (HTH) motif in the N-terminus (Xie et al., 2018). HTH motifs are commonly found in transcription factors as nucleic acid binding domains (Aravind et al., 2005). With the addition of the N-terminal HTH DNA binding motif, PtrEPSP-TF exhibited nuclear accumulation and functioned as a transcriptional repressor. By comparing 57 EPSP synthase isoforms from 42 plant genomes, the HTH motif was found to be almost entirely missing in EPSP synthases in non-vascular, algal, and monocots, but was found in many dicots (Xie et al., 2018). The presence of secondary cell wall is a key distinguishing feature separating dicots from algae and mosses. It is intriguing that this shikimate pathway derived-EPSP synthase isoform appears to have obtained a regulatory function modulating the expression of processes that are ubiquitous in dicots relative to other plants. With this in mind, we hypothesized that domain co-option may have occurred during the course of evolution when early dicotyledonous plants attained complex cell wall structure (Weng et al., 2008a; Tohge et al., 2013). The discovery of the additional regulatory loop of *MYB46* in *Populus* also supports the existence of woody plant-specific regulatory mechanisms in lignin biosynthesis.

## Perspectives on the Origin and Evolution of Lignin Biosynthesis in Plants

The phenylpropanoid pathway produced thousands of metabolites which are essential for plant terrestrialization and subsequent radiation. Lignins appeared as specialized metabolites with the evolution of tracheophytes. The identification of progenitors of lignin biosynthetic genes in bryophytes provides new insights into the origin of lignin biosynthesis (Kriegshauser et al., 2021). The recent progress on genome sequencing of Charophyte algae, bryophytes, lycophytes, and ferns have also provided unprecedented opportunities to study the origin of phenylpropanoid biosynthetic pathway (Szövényi et al., 2021).

On the basis of current knowledge of lignin biosynthetic pathways across tracheophytes, we conclude that most lignin biosynthetic genes experienced expansions and neofunctionalization. As a result, lignin biosynthetic pathway has become increasingly complex evidenced by the existence of many alternate pathways and regulatory hierarchies. In support of this hypothesis many of the alternative pathways have been shown to be lineage specific. Lignin biosynthesis in monocots served an example of diversification. For example, PTAL-mediated by-pass route in lignin biosynthesis and PMT-mediated lignin modification are specific to monocots (Petrik et al., 2014; Barros et al., 2016). Equally, S-lignin biosynthesis in *S. moellendorffii* suggested that S-lignin biosynthetic pathway may be evolved multiple times or lost in gymnosperms and other pteridophytes (Weng and Chapple, 2010).

Transcriptional regulatory modules have been shown to be generally conserved for phenylpropanoid and lignin biosynthesis; however, a third layer of MYB TFs are not evolutionarily conserved and have witnessed a wide expansion of family members. Finally, newly identified TFs, such as EPSP-TF, have been shown to regulate lignin biosynthesis specifically in woody plants (Xie et al., 2018). The studies on transcriptional regulation of lignin biosynthesis represents an emerging opportunity to understand the phylogenetic occurrence of the phenylpropanoid pathway and lignin biosynthesis in plants.

## Concluding Remarks and Future Directions

In this review, we summarized the phylogenetic occurrence of lignin biosynthetic genes and related transcriptional regulation across different plant species. Comprehensively, the core enzymes in lignin biosynthesis and basal transcriptional regulatory module are conserved among embryophytes, although bryophytes do not produce lignin. With evolutionary time, lignin composition diversity has increased and has been associated with gene duplication, functional gene co-option, and neo- and sub-functionalization, which involved many structural genes and transcriptional regulators. In addition, concomitant with the increase of lignin biosynthetic complexity, is the increase in functional diversity, e.g., water conductivity and defense. As most of the current knowledge of lignin biosynthesis is based on the study of a few angiosperms, identification and functional characterization of the lignin biosynthetic pathways and their regulation in lower plants will provide a comprehensive view of their evolutionary history and lead to new insights in lignin biosynthesis.

## Author Contributions

TY and KF drafted the manuscript. MX, JB, TT, GT, WM, and J-GC revised the manuscript. All authors contributed to the article and approved the submitted version.

## Conflict of Interest

The authors declare that the research was conducted in the absence of any commercial or financial relationships that could be construed as a potential conflict of interest.

## Publisher’s Note

All claims expressed in this article are solely those of the authors and do not necessarily represent those of their affiliated organizations, or those of the publisher, the editors and the reviewers. Any product that may be evaluated in this article, or claim that may be made by its manufacturer, is not guaranteed or endorsed by the publisher.
